# A Secure Partial RFID Ownership Transfer Protocol with Multi-Owners

**DOI:** 10.3390/s20010022

**Published:** 2019-12-19

**Authors:** Jia-Ning Luo, Ming-Hour Yang

**Affiliations:** 1 Department of Information and Telecommunications Engineering, Ming-Chuan University, Taoyuan 33350, Taiwan; 2 Department of Information and Computer Engineering, Chung Yuan Christian University, Taoyuan 32023, Taiwan

**Keywords:** mobile radio frequency identification (RFID), ownership transfer

## Abstract

Mobile radio frequency identification (RFID) has been extensively applied in a wide range of fields. In supply chain management, RFID is used to more efficiently manage the ownership transfer of cargo. The transfer of a group of tags belonging to multiple owners is often required at the front end of a supply chain. This study, therefore, proposes a secure, high-performance threshold multi-owner partial tag ownership transfer protocol that supports a mobile RFID environment and features the capabilities and security required for supporting existing ownership transfer environments (e.g., application for different authorities, designation of the transfer target, and ownership transfer of a group of tags). Moreover, the proposed protocol can resist against most of the known attacks on RFID.

## 1. Introduction

Mobile radio frequency identification (RFID) is a combination of a wireless network, mobile telecommunication technology, and RFID systems [1,2]. Mobile RFID is characterized by simple computing power, storage capacity, and the simultaneous reading and writing of multi-tag information. These features facilitate product identification and follow-up management. Mobile RFID is widely applied in supply chain management, access control, bill payment, smart home development, military supply control, and health care medication administration. Because RFID can effectively manage the flow and processing of goods, large-scale retail chain stores such as Walmart have saved roughly $1.4 billion USD in cost by using RFID technologies [1].

RFID has become an integral part of supply chain management in recent years and has been continually advancing and becoming more affordable. Therefore, various components of a supply chain, including raw material supplier, product manufacturer, wholesaler, retailer, and end consumer, can employ RFID for follow-up management. Manufacturers use RFID tags to identify goods information and conduct inventory. Retailers use RFID tags to keep track of and manage product information and provide consumers with a convenient shopping platform and various services. Consumers use RFID tags to obtain product and post-sale information. To facilitate the management of supply chain automation and effectively engage in product ownership transfer [3,4,5,6,7,8,9], products labeled with RFID tags undergo multiple ownership transfers throughout their life cycle from their introduction to the decline stage.

For secure transfer of product ownership, designation must be ensured while also avoiding the windowing problem (i.e., new and old owners simultaneously owning a tag) and providing forward and backward secrecy. Forward secrecy means that the new owner cannot identify and decrypt messages that were transmitted between the tag and its old owner. Backward secrecy means that the old owner cannot receive and decrypt the messages transmitted between the tag and its new owner. In the process of tag ownership transfer, since a tag has limited computing power and an RFID system employs wireless transmission, hackers can access messages sent by the tag or reader. The RFID system may also suffer from security threats, such as message modification, replay attack, man-in-the-middle attack, tracking attack, denial-of-service (DoS) attack, and a counterfeit tag reader attack.

When products are at the end of a supply chain, each product owner usually owns only a small number of products. Therefore, owners perform ownership transfer only once for a small number of products. Osaka et al. [6] proposed an ownership transfer method for a single tag in such an application environment in which few tags are being transferred. However, their method is associated with security flaws. For example, update-key messages are vulnerable to modification attacks, which causes an asynchronous service, compromised forward secrecy, and the windowing problem during the transfer period [9,10]. Jäppinen et al. [11] verified the integrity of update-key messages to reduce the likelihood of asynchronous communication between tags and end servers, but the methods for doing so still engender vulnerability to attacks, which results in persistent asynchronous problems [12]. Hence, Yoon et al. [13], Chen et al. [14], Yang et al. [15], and Dimitriou et al. [16] proposed new ownership transfer methods to address the asynchronous service problem and ensure the forward secrecy of ownership transfer. However, the protocol presented by Dimitriou et al. [16] is vulnerable to counterfeiting and replay attacks [17]. The methods developed by Yoon et al. [13] and Chen et al. [14] are also associated with security concerns such as lack of support for backward secrecy, the inability to ensure location privacy, and windowing problems [9,18]. Yang et al. [19] proposed layered object transport protocol (LOTP), which is applicable for environments employing mobile RFID. LOTP [19] involves the transfer of tag ownership through a trusted third party (TTP) to overcome attacks that occur during ownership transfer through mobile RFID. However, LOTP can only transfer one tagged object at a time and cannot efficiently transfer a large number of tagged objects.

Therefore, when products are at the front end of a supply chain, manufacturers or wholesalers generally perform a single transfer of ownership for an extremely large number of products. However, a protocol causes problems due to inefficiency if it can only transfer the ownership of a single tag. Zuo [12] and He et al. [20] used group keys to simultaneously authenticate and transfer the ownership of all tags in a group. However, Zuo’s protocol resulted in denial of service (DoS) attack when the updated key was subject to a desynchronization attack. Subsequently, Jannati et al. [21] proposed a solution to the DoS problem caused by a modification to update-key messages. However, all of these group transfer protocols have a limitation of only being able to perform a single transfer for all tags of an owner and not being able to perform a partial transfer of only some tags in a group. In other words, the flexibility in object ownership transfer is limited, which renders relevant methods impractical. Therefore, Molnar et al. [22] proposed using a split back-end server and a tree of secrets shared between a large number of tags to achieve partial ownership transfer. The number of nodes in the tree represents the number of times that a reader is authorized to read a tag after a binary tree has been transferred from the reader to the back-end server. The new owner can achieve partial ownership transfer by obtaining the tree of secrets of a tag through the back-end server. Tsai et al. [23] proposed an ownership transfer method with grouping the proof protocol that allows for grouping proof and partial ownership transfer of tag groups while ensuring the integrity of the tagged cargo. Yang et al. proposed a tag group ownership transfer protocol with a trust third party (TTP) [24] and without a TTP [25]. This protocol generates a key for a tree of partial group communication by employing the group communication key shared between the tags and server to achieve partial ownership transfer of tag groups. In addition, this protocol can resist most known attacks and protect and secure the privacy of owners.

Ownership transfer is frequently required for a large number of products, particularly when the raw materials or goods of upstream industries are distributed along the supply chain. These raw materials and goods generally belong to different owners and are simultaneously loaded onto the same cargo ship or cargo truck. However, existing methods for secure tag group ownership transfer are limited to only the transfer of objects of a single owner and are not applicable to ownership transfer for multi-object owners. Hence, Kapoor et al. [26] proposed a multi-owner ownership transfer method. However, their method is vulnerable to DoS attacks because it places the tag and server key in a desynchronized state when the key is updated. Moreover, their protocol can only transfer the ownership of a single tag of multiple owners. The transfer efficiency is reduced when transferring the ownership of multiple tags because each tag must independently perform all steps to authenticate the updated key, which increases the information and calculation load. To address this problem, Sundaresan et al. [27] proposed a multi-owner/multi-tag ownership transfer method that uses a group secret value shared between the owners and a group of tags to generate acknowledgments for every tag that must be transferred and send the acknowledgments to all tags in the group. Because each tag group that is designated for transfer generates a message based on its tag identification number (ID), each tag must examine every message received for a tag ID to acknowledge that its tag ID is contained in the transfer of this tag group to simultaneously partially transfer the ownership of tag groups of the owners. However, because the owners in a group use a shared secret to protect the tag message, the method cannot protect the data privacy of the owners in that group. Subsequently, Sundaresan et al. proposed another approach for protecting group communication privacy by applying different group secret values to each owner and tag group [28]. However, in both methods, the process of ownership transfer requires each tag in a group to compute the message for each tag that needs to be transferred in order to acknowledge that its tag needs to be transferred. For example, if a group containing 2000 tags needs to transfer 1000 tags to a new owner, then these 2000 tags must acknowledge that the 1000 messages contain its tag ID. Therefore, each tag requires a large amount of information, a high calculation load, and long transfer time. In addition, the two previously mentioned methods of ownership transfer for multi-owner and partial tag group environments are vulnerable to attacks (e.g., replay, tracking, or DoS attacks) and do not achieve forward secrecy [29]. Table 1 summarized the categories of RFID ownership transfer protocols.

This study proposes a secure, high-performance multi-owner partial ownership transfer protocol to overcome the problems concerning the performance of existing multi-owner tag ownership transfer methods and address the security threats and privacy concerns that may arise in the process of ownership transfer. In this proposed multi-owner ownership transfer protocol, the old owners and new owners of a tag group may differ regarding their jurisdiction. In our protocol, the permissions of several owners are required to transfer a tag or multiple tags from a group of owners to the others. A single user cannot transfer his/her ownership of the tags to others. Our protocol is useful in the supply chain management. The factory assigns ownership of tags to a group of employees. However, it is not necessary to obtain the consent of each employee when transferring ownership. When one of the old owners initiates ownership transfer, a threshold scheme is used to ensure that (1) the consent of a certain number of old owners is obtained before the owner can partially transfer the ownership of a tag group to new owners, and (2) the proposed method can resist most of the known attacks and offer most of the security and privacy protection properties for ownership transfer. This study makes the following contributions to the literature. The multi-owner multi-tag partial ownership transfer protocol (1) is applicable in a mobile RFID environment, (2) can transfer the ownership of one, some, or all tags, (3) provides two-way authentication between a tag, reader, and a back-end server, (4) ensures that tag ownership is only transferred to the designated owners, (5) is secure and immune to replay attacks, eavesdropping, message modification attacks, and tracking attacks (i.e., protects owner privacy) and provides forward and backward secrecy. Lastly, it (6) features high performance that is not related to the reader participating in the transfer or the number of tags and does not increase information and calculation load considerably when the number of owners and tags increases.

This paper is organized as follows. Section 2 introduces the environmental assumptions of the proposed ownership transfer protocol and the relationships among the tag, reader, and the back-end server. Section 3 provides a detailed description of the proposed protocol. Section 4 compares and analyzes the security of the proposed method and other RFID ownership transfer methods. Section 5 presents the calculation performance of the proposed protocol for analysis and a comparison with those introduced in relevant studies, and, lastly, Section 6 concludes this study.

## 2. Multi-Owner Multi-Tag Ownership Transfer Method

**Figure 1:**
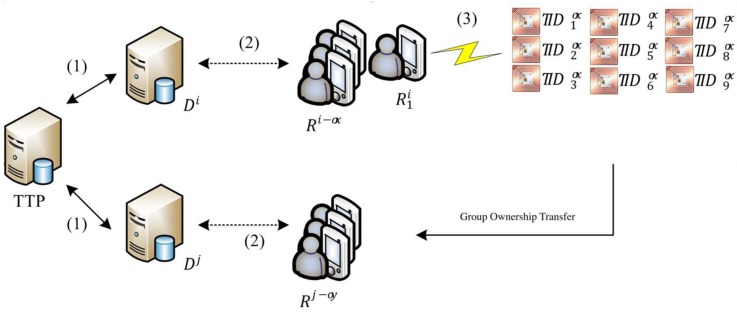
Multi-owner ownership transfer architecture.

This study proposes a secure multi-owner multi-tag ownership transfer method for a mobile RFID network environment. Figure 1 illustrates the architecture of the proposed system. The mobile device of a member of the old owner group sends a signal for ownership transfer, reads the partial tags in the group to be transferred, and sends the transfer message collectively generated by these tags to a back-end server. The server then notifies the group of old owners. If the partial signature of *n* old owners who consent to partial tag ownership transfer exceeds the threshold value, then the group of old owners and the group of new owners jointly conduct ownership transfer.

### 2.1. System Architecture

The ownership network architecture in Figure 1 reveals that the servers, mobile readers, and tags have different computing capabilities. Connection security is discussed in three parts marked by (1), (2), and (3) in Figure 1. Connection (1) in Figure 1 shows that the server exhibits a computing capability sufficiently powerful to support existing encryption algorithms such as the Advanced Encryption Standard (AES) and Rivest–Shamir–Adleman (RSA). Therefore, the proposed protocol employs existing encryption methods to ensure secure communication between the servers. Connection (2) in Figure 1 indicates that the mobile readers engage in two-way communication with other mobile readers by using existing mobile communication technologies or wireless network technologies and back-end servers. Extant security communication technologies such as the X.509 security architecture of telecommunication networks or IEEE802.11i are employed to protect the transfer security of intermediate messages. Connection (3) in Figure 1 reveals that, because tags have limited computing capability, lightweight cryptography methods such as the data encryption standard lightweight [30] or Grain [31] should be used when communicating with mobile readers to secure the messages communicated between the tag and mobile reader. Because the environment involved in part (3) is the riskiest, the present study assumes that the network environment in connection (3) is an insecure communication environment that is vulnerable to attacks. To provide a detailed description of the proposed ownership transfer method, the ownership transfer environment of the tag management service is assumed to be characterized by four properties.

The first property is that a mobile reader connects only to a single back-end server during communication to manage the tags and that the back-end server under the authority of the mobile reader to which ownership is transferred in and out is likely the same back-end server or a back-end server under different authority. In the set $\;R^{i}\;$ of *a* readers under the authority of back-end server $\;D^{i}\;$ with ID $DID^{i}$, any one of the readers $\;R_{a}^{i}\;$ with independent ID $RID_{a}^{i}\;$ must satisfy the relation in Equation (1).

$R^{i}=\left\{R_{1}^{i},R_{2}^{i},\ldots ,R_{a}^{i}\right\}$ where $R^{i}$ is under the authority of $D^{i}$ and $R^{j}$ is under the authority of $D^{j}$, which satisfies the equation below.

(1) $$\forall i,j\;R^{i}\cap^{\;}R^{j}=\emptyset \;\mathrm{and}\;iff\;i\neq j;\;\mathrm{otherwise},\;R^{i}\cap^{\;}R^{j}=R^{i}$$

The second property is that the set $\;R^{i}\;$ of readers under the back-end server $\;D^{i}\;$ consists of a set $\;R_{}^{i-ox}\;$ of readers owned by *p* owners, and, in this multi-owner set $\;R_{}^{i-ox}$, all of the owners have collective ownership to the tags in group $\;G_{0}^{ox}$. Without loss of generality, an ownership transfer protocol implemented through reader $\;R_{1}^{i}\;$ must satisfy the relation in Equation (2).

(2) $$R_{}^{i-ox}=\left\{R_{1}^{i},R_{2}^{i},\ldots ,R_{p}^{i}\right\},\;R_{}^{i-ox}\subseteq R^{i},\;satisfying\;\forall ox,oy\;G_{0}^{ox}\cap^{\;}G_{0}^{oy}=\emptyset$$

$$iff\;ox\neq oy,\;otherwise\;G_{0}^{ox}\cap^{\;}G_{0}^{oy}=G_{0}^{ox}$$

**Figure 2:**
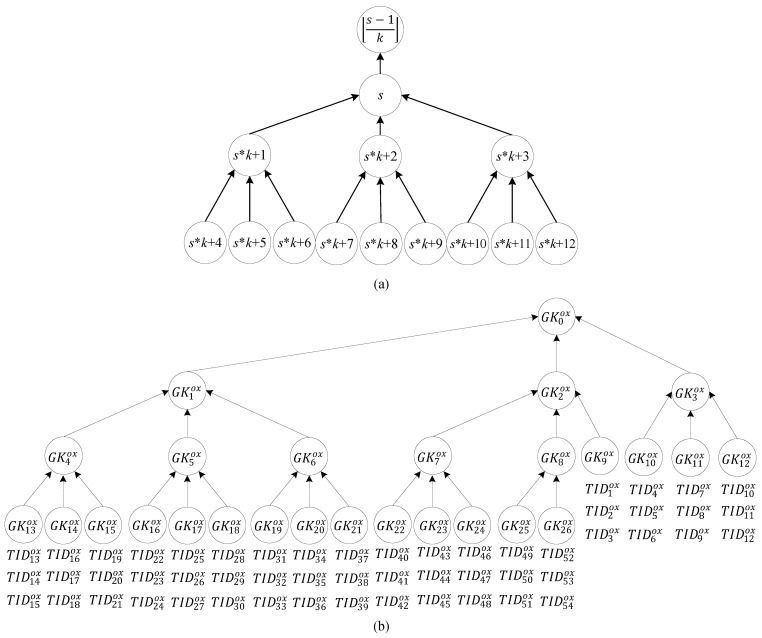
(**a**) Tags naming rule (**b**) Tree of tag group key.

The third property is that v tags in multi-owner set $R_{}^{i-ox}$, which is managed by the back-end server $D^{i}$, belong to group $G_{0}^{ox}$, with the group key defined as $GK_{0}^{ox}$. The server splits the tag groups into a k-ary tree of group keys, which generates a key tree with the height $h_{max}=[log_{k}\left(\frac{v}{k}\right)]$. Next, the sequence of the k-ary group ID moves from top to bottom and from left to right. The parent node $G_{\left[\frac{s-1}{k}\right]}^{ox}$, the children nodes $G_{s*k+1}^{ox}$ through to $G_{s*k+k}^{ox}$, and their relationship with the rules are shown in Figure 2a. Group key $GK_{1}^{ox}$ can encrypt the group message to $TID_{13}^{ox}$ through to $TID_{39}^{ox}$, and tag IDs $TID_{1}^{ox},\;TID_{2}^{ox},\;\mathrm{and}\;TID_{3}^{ox}$ can use the keys $Kt_{1}^{ox},Kt_{2}^{ox},\;\mathrm{and}\;Kt_{3}^{ox}$ shared with the server to decrypt the group message that is encrypted by group key $GK_{9}^{ox}$. Therefore, the node with the group ID $G_{s}^{ox}$ is composed of one to k subtrees, and the group key containing any node $G_{s}^{ox}$ is expressed in Equation (3). The group key $GK_{s}^{ox}$ consists of a parent group $G_{spar}^{ox}$ containing $G_{s}^{ox}$, the intersection of $G_{s}^{ox}$ with $G_{spar}^{ox}$ equals $G_{s}^{ox}$, and the intersection of the difference between $G_{0}^{ox}$ and $G_{spar}^{ox}$ with $G_{s}^{ox}$ is the empty set, as shown in Equation (4).

(3) $$G_{s}^{ox}=\left\{GK_{l}^{ox}|\forall l\;GK_{l}^{ox}\in G_{s}^{ox},sk^{h}+\frac{k^{h}-1}{k-1}\leq l\leq sk^{h}+\frac{k\left(k^{h}-1\right)}{k-1},h\in {\mathbb{Z}}_{0}^{+},s\in {\mathbb{Z}}_{0}^{+}\right\}$$

(4) $$G_{spar}^{ox}=\left\{GK_{s}^{ox}\in G_{\left[\frac{s-\frac{k^{h}-1}{k-1}}{k^{h}}\right]}^{ox},\;h\in {\mathbb{Z}}_{0}^{+},\;s\in {\mathbb{Z}}_{0}^{+}\right\},$$

$\forall s\;GK_{s}^{ox}\;\mathrm{is}\;\mathrm{the}\;\mathrm{authority}\;\mathrm{of}\;R_{}^{i-ox},\;\mathrm{satisfying}\;G_{s}^{ox}\cap^{\;}G_{spar}^{ox}=G_{s}^{ox}\mathrm{and}\;\left(G_{0}^{ox}-G_{spar}^{ox}\right)\cap^{\;}G_{s}^{ox}=\emptyset$

The fourth property is the leaf group that is defined as the leaf node with IDs [(vk)−1k−1] through [(vk)−1k−1]+[vk]−1 on $\left[\frac{v}{k}\right]$ key trees connected to the tags, as shown in Equation (5). For example, tag IDs $TID_{1}^{ox},\;TID_{2}^{ox},\;\mathrm{and}\;TID_{3}^{ox}$ are connected to the leaf node $G_{leaf,1}^{ox}$ with group ID $G_{9}^{ox}$.

(5) $$G_{leaf,m}^{ox}=\left\{TID_{l}^{ox}|\forall l\;TID_{l}^{ox}\in G_{leaf,m}^{ox},\;\left(m-1\right)k+1\leq l\leq mk,1\leq m\leq [\frac{v}{k}]\right\}$$

**Figure 3:**
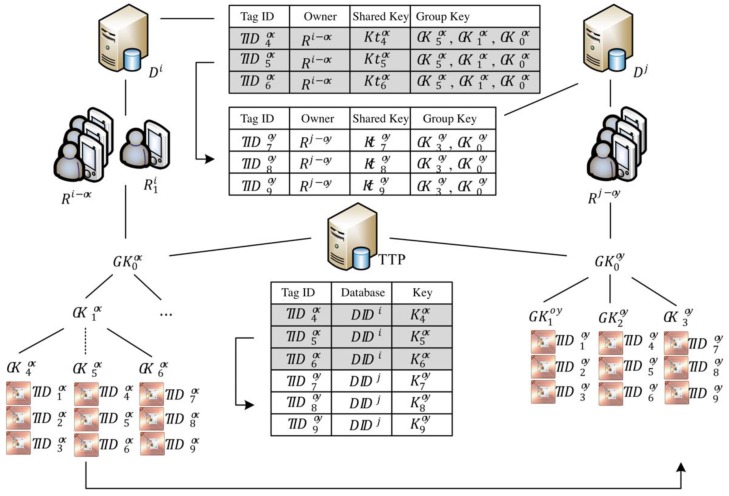
Example of tag group transfer IDs.

Figure 3 presents an example in which an owner’s reader $R_{1}^{i}$ intends to transfer the ownership of tag IDs $TID_{4}^{ox},TID_{5}^{ox},\mathrm{and}\;TID_{6}^{ox}$ in tag group $G_{5}^{ox}$ (indicated by the dashed line in the middle of the key tree) from the owner group $R_{}^{i-ox}$ under the authority of $D^{i}$ to the owner group $R_{}^{j-oy}$ under the authority of $D^{j}$. If the owner’s reader $R_{1}^{i}$ obtains consent from the group of old owners through the server $D^{i}$ of the old owner group to partially transfer tags, then server $D^{i}$ uses the communication key shared between $D^{i}$ and the tags on the topmost level in Figure 3 to generate and send the ownership transfer message to a trust third party (TTP). Subsequently, the TTP sends an update-key message to multi-owner sets $R_{}^{i-ox}$ and $R_{}^{j-oy}$ and tag group $G_{0}^{ox}$ to simultaneously update the key and avoid the windowing problem. As indicated on the right of Figure 3, because the back-end server $D^{j}$ has authority over its own key tree, when tag IDs $TID_{4}^{ox},TID_{5}^{ox},$ and $TID_{6}^{ox}$ are transferred to multi-owner sets $R_{}^{j-oy}$, the owner inserts tag IDs $TID_{7}^{oy},TID_{8}^{oy},\;\mathrm{and}\;TID_{9}^{oy}$ in tag group $G_{3}^{oy}$ on the far right, and these IDs are not necessarily the same as the output tag IDs. Lastly, the TTP changes the shared keys of tags $TID_{4}^{ox},TID_{5}^{ox},$ and $TID_{6}^{ox}$ under $D^{i}$, as shown at the bottom of Figure 3, to a shared management key of tags $TID_{7}^{oy},TID_{8}^{oy},\mathrm{and}\;TID_{9}^{oy}$ under $D^{j}$. Details of the ownership transfer protocol are provided in Section 3.

### 2.2. Transfer of Multiple Tag Groups

Tags to be transferred may belong to different groups. In other words, the transfer of ownership of all the tags to be transferred cannot be achieved by transferring the tags of a single group. In this case, the proposed multi-owner multi-tag ownership transfer method is implemented multiple times to solve this problem. As indicated in Figure 2b, because the tags $TID_{40}^{ox}–TID_{42}^{ox}$, $TID_{46}^{ox}–TID_{48}^{ox}$, and $TID_{52}^{ox}–TID_{54}^{ox}$ belong to different groups and the transfer of ownership of all of the tags cannot be achieved by transferring the tags of a single group. Transferring these tags requires the ownership of tag groups $G_{22}^{ox}$, $G_{24}^{ox}$, and $G_{26}^{ox}$ to be transferred three times. The owners’ readers are activated to send the ownership transfer request (OT) of groups IDs $G_{22}^{ox}$, $G_{24}^{ox}$, and $G_{26}^{ox}$ to the tags. The ownership transfer of group IDs $G_{22}^{ox}$, $G_{24}^{ox}$, and $G_{26}^{ox}$ can be simultaneously conducted by implementing the proposed protocol multiple times.

## 3. Multi-Owner Multi-Tag Ownership Transfer Protocol

### 3.1. Initialization

Table 2 lists the notations in this paper. In our protocol, consent must be obtained from the majority of owners of the reader set $R_{}^{i-ox}$ before ownership transfer. Therefore, the threshold signature scheme presented by Harn [32] is used to confirm majority consent before proceeding to transfer ownership. When *n* owners receive a transfer request, the partial signatures of only *t* consenting owners are required and are sent to the server for grouping and verification. If the partial signatures match the ownership transfer message, then most owners consented to tag ownership transfer. The next three steps are described as follows.

The first step is the group key and the secret key generation stage. This step must be completed before ownership transfer. Each owner is allocated a secret key for partial signature generation, a verification key for partial signature generation, and a verification key for group signature generation. 

The second step is the partial signature generation stage. When one of the owners of the reader set $R_{}^{i-ox}$ sends an ownership transfer request message (OT), the server asks other owners whether they consent to ownership transfer. Consenting owners send the partial signature generated by the request message to the server for verification.

The third step is the group signature verification stage. The server conducts verification and grouping after receiving the partial signature and then verifies whether the group signature matches the OT message. If the message matches, then most owners agreed to proceed with the ownership transfer.

### 3.2. Ownership Transfer Request

**Figure 4:**
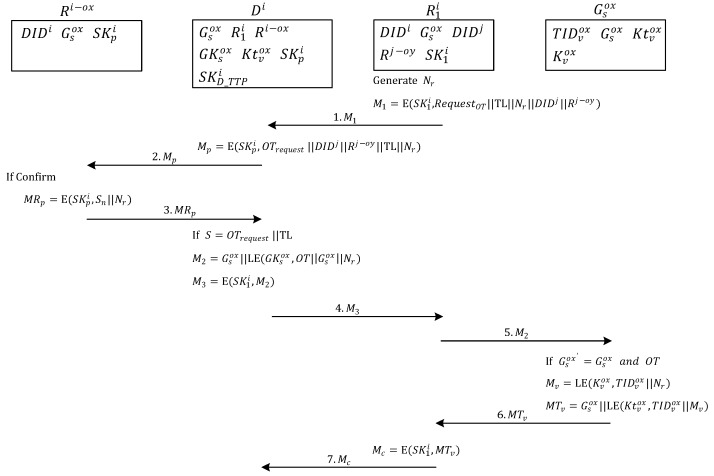
Ownership transfer request.

Figure 4 shows the selected reader wants to get the ownership transfer permission from the original owners. In Step 1, the reader $R_{1}^{i}$ belonging to one of the owners in the multi-owner set $R^{i-ox}$ of tag group $G_{s}^{ox}$ expresses intent to transfer the ownership of the object represented by each tag in the tag group $G_{s}^{ox}$ to the owners in the multi-owner set $R^{j-oy}$. First, owner $R_{1}^{i}$ generates a random number $N_{r}$ and uses the key $SK_{1}^{i}$ to encrypt an OT, tag list (TL) of all the tags to be transferred, pseudo-random number $N_{r}$, server ID $DID^{j}$ of the transfer target, and multi-owner set $R^{j-oy}$ of the new owner. After encryption, message $M_{1}$ is generated and sent to the management server $D^{i}$ of $R_{1}^{i}$ requesting $D^{i}$ to ask other owners whether they consent to ownership transfer.

In Step 2, after server $D^{i}$ receives $M_{1}$, it decrypts the message by using the key $SK_{1}^{i}$ shared with owner $R_{1}^{i}$, and confirms that owner $R_{1}^{i}$ wants to transfer the tags in the TL. Server $D^{i}$ encrypts the OT, server ID $DID^{j}$ of the transfer target, multi-owner set $R^{j-oy}$ of the new owner, TL of all the tags to be transferred, and pseudo-random number $N_{r}$ by using the key $SK_{p}^{i}$, which is shared between server $D^{i}$ and each owner in the multi-owner set $R^{i-ox}$. Lastly, the server sends message $M_{p}$ to each owner in the multi-owner set $R^{i-ox}$ asking whether they consent to the ownership transfer.

In Step 3, when each owner in the multiowner set $R^{i-ox}$ with the ownership of tag group $G_{s}^{ox}$ receives $M_{p}$, the message $M_{p}$ is decrypted using the key $SK_{p}^{i}$, which is shared between server $D^{i}$ and each owner. The consent of the owner to the ownership transfer is verified. If consent is provided, then the key $SK_{p}^{i}$ shared between server $D^{i}$ and each owner is used to encrypt the partial signature $S_{n}$ and pseudo-random number $N_{r}$, which generates the message $MR_{p}$. This is then sent to server $D^{i}$.

In Step 4, server $D^{i}$ collects the message $MR_{p}$ from owners in the multi-owner set $R^{i-ox}$ and uses the key $SK_{p}^{i}$, which is shared between server $D^{i}$ and owners, to decrypt message $MR_{p}$ and perform the verification of partial signature $S_{n}$ to check whether it matches the signature message $OT_{request}||TL$. If the signature matches, then most owners consented to ownership transfer and the protocol proceeds with the ownership transfer. If the collected partial signatures are not enough, or the signature does not match, then owners did not consent to ownership transfer and the protocol terminates the ownership transfer.

When most owners provide consent to proceed with ownership transfer, server $D^{i}$ first identifies the communication key $GK_{s}^{ox}$ of the tag group in the TL. Next, it uses the group communication key $GK_{s}^{ox}$ to encrypt the OT (approved by the owners in the multi-owner set $R^{i-ox}$), group $G_{s}^{ox}$ of tags to be transferred, the pseudo-random number $N_{r}$, and group ID $G_{s}^{ox}$, subsequently generating message $M_{2}$. Message $M_{2}$ is then encrypted using the communication key $SK_{1}^{i}$, which is shared with the owner, to produce message $M_{3}$, which is transmitted to owner $R_{1}^{i}$.

In Step 5, after owner $R_{1}^{i}$ receives message $M_{3}$, it decrypts the message by using the communication key $SK_{1}^{i}$ shared with server $D^{i}$. Then the extracted message $M_{2}$ is sent to tag group $G_{s}^{ox}$ as a broadcast message.

In Step 6, when any tag $TID_{v}^{ox}$ in tag group $G_{s}^{ox}$ receives message $M_{2}$, the tag confirms whether the group tag ID in the message matches the ID $G_{s}^{ox}$ of the tag group to which it belongs. After confirming that it belongs to the tag group $G_{s}^{ox}$, each tag uses the communication key $Kt_{v}^{ox}$ shared with server $D^{i}$ to decrypt the OT (approved by owners in the multi-owner set $R^{i-ox}$) and then reconfirms that the tag group $G_{s}^{ox}$ to be transferred matches the ID of the tag group $G_{s}^{ox}$ to which it belongs. If the IDs match, then the tag uses the management key $K_{v}^{ox}$ shared with the TTP to encrypt its tag ID $TID_{v}^{ox}$ and the pseudo-random number $N_{r}$ from owner $R_{1}^{i}$, which produced the authentication message $M_{v}$. Next, the tag uses the communication key $Kt_{v}^{ox}$ shared with server $D^{i}$ to encrypt tag ID $TID_{v}^{ox}$, pseudo-random number $N_{r}$, and its tag group ID $G_{s}^{ox}$, which generates message $MT_{v}$ and is sent to owner $R_{1}^{i}$.

In Step 7, after owner $R_{1}^{i}$ receives message $MT_{v}$, it decrypts the message by using the communication key $SK_{1}^{i}$ shared with server $D^{i}$, subsequently producing message $M_{c}$, which is then sent to the management server $D^{i}$ of $R_{1}^{i}$.

### 3.3. Authentication of Tags and Transfer of Ownership

**Figure 5:**
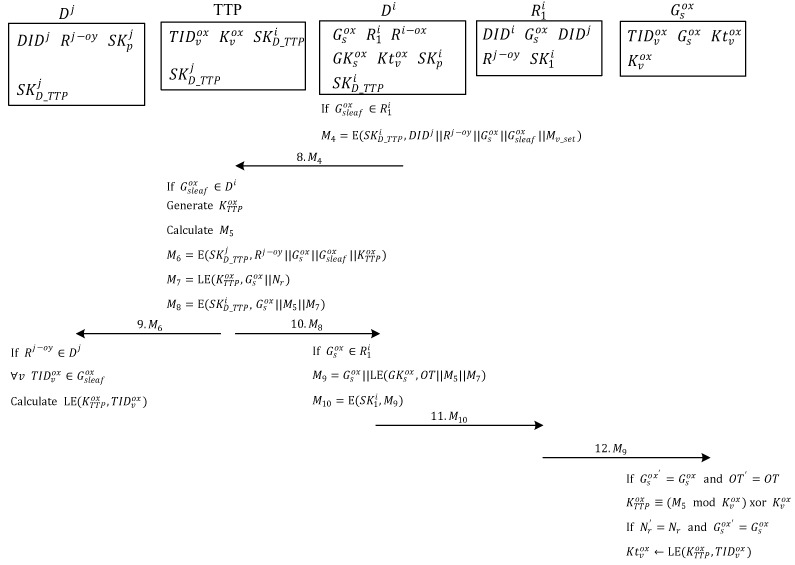
Authentication of tags and transfer of ownership.

Figure 5 shows the authentication of tags and the ownership transfer process. In Step 8, because tag group $G_{s}^{ox}$ may contain more than one tag, server $D^{i}$ must collect message $M_{c}$ sent by all of the tags in tag group $G_{s}^{ox}$. Subsequently, because tag group $G_{sleaf}^{ox}$ is a set of all leaf nodes of the group ID subtree of group $G_{s}^{ox}$, $G_{sleaf}^{ox}$ comprises all of the tags in tag group $G_{s}^{ox}$. When server $D^{i}$ receives message $M_{c}$, the server decrypts each message $M_{c}$ by using the key $SK_{1}^{i}$ shared with owner $R_{1}^{i}$ to extract $MT_{v}$ and then decrypts each message $MT_{v}$ by using the secret value $Kt_{v}^{ox}$ shared between server $D^{i}$ and each tag to compare the tag ID with the authentication tag. Next, the server performs comparisons to determine whether all of the tag IDs in tag group $G_{sleaf}^{ox}$ are consistent with the tag IDs received, checks whether all tags in tag group $G_{sleaf}^{ox}$ are available, and checks whether $R_{1}^{i}$ has ownership of tag group $G_{s}^{ox}$.

Server $D^{i}$ uses the communication key $SK_{D\_TTP}^{i}$ shared with a TTP to encrypt the server’s identity $DID^{j}$ of the transfer target, multi-owner set $R^{j-oy}$ of the new owner, tag group $G_{s}^{ox}$, and tag group $G_{sleaf}^{ox}$, which consists of the IDs of all the tags to be transferred. $M_{v\_set}$ of message $M_{v}$ is transmitted by all of the tags in tag group $G_{sleaf}^{ox}$. $M_{v\_set}$ is defined in Equation (6). Subsequently, message $M_{4}$ is generated and transmitted to the TTP, which requests that the TTP use the management key shared between the TTP and the tags to update the communication key on the tag for completing the ownership transfer.

(6) $$M_{v\_set}^{\;}=\left\{\mathrm{LE}(K_{v}^{ox},TID_{v}^{ox}||N_{r}^{\;})│\;\forall v\;TID_{v}^{ox}\in G_{sleaf}^{ox}\right\}$$

In Steps 9 and 10, after the TTP receives message $M_{4}$, the communication key $SK_{D\_TTP}^{i}$ shared with server $D^{i}$ is used to decrypt message $M_{4}$. After confirming that the tag group $G_{sleaf}^{ox}$ belongs to server $D^{i}$, the TTP uses the management key $K_{v}^{ox}$ shared between each tag and the TTP to decrypt each tag message $M_{v}$ in the $M_{v\_set}$. The TTP then extracts tag ID $TID_{v}^{ox}$ to authenticate the tag and determine whether each tag ID in tag group $G_{sleaf}^{ox}$ within message $M_{4}$ matches the tag IDs in $M_{v\_set}$ and checks whether the pseudo-random number is the same for all tag messages $M_{v}$. If all of them match, then the TTP randomly generates a new secret value $K_{TTP}^{ox}$ and uses the Chinese remainder theorem [33] in Equation (7) to calculate message $M_{5}$.

(7) $$M_{5}\equiv \overset{k_{\;}^{h_{max}^{\;}–\log_{k}^{r}}}{\underset{s=1}{\sum }}\left(\left(K_{TTP}^{ox}\;\mathrm{xor}\;K_{s}^{ox}\right)*m_{s}^{ox}*{m^{\prime }}_{s}^{ox}\right)\;\left(\mathrm{mod}\;M\right),\;\mathrm{where}\;M=\overset{k_{\;}^{h_{max}^{\;}–\log_{k}^{r}}}{\underset{s=1}{\prod }}K_{s}^{ox},$$

$$m_{s}^{ox}=\frac{M}{K_{s}^{ox}},{m^{\prime }}_{s}^{ox}*m_{s}^{ox}\equiv 1\;\left(\mathrm{mod}\;K_{s}^{ox}\right)$$

The TTP encrypts the multi-owner set $R^{j-oy}$ of the new owner, tag group $G_{s}^{ox}$, and tag group $G_{sleaf}^{ox}$, which consists of the IDs of all of the tags to be transferred, and the secret value $K_{TTP}^{ox}$ into message $M_{6}$ by using the communication key $SK_{D\_TTP}^{j}$ shared between the TTP and server $D^{i}.$ Next, the random number $N_{r}$ and $G_{s}^{ox}$ are encrypted into message $M_{7}$ by using the secret value $K_{TTP}^{ox}$. $M_{5}$, $M_{7}$, and $G_{s}^{ox}$ are encrypted into message $M_{8}$ by using the communication key $SK_{D\_TTP}^{i}$ shared between the TTP and server $D^{i}.$ Messages $M_{6}$ and $M_{8}$ are then sent to the new owners and the old owners for ownership transfer.

After server $D^{i}$ of the new owners receives messages $M_{6}$, it decrypts the message by using the communication key $SK_{D\_TTP}^{j}$ shared with the TTP. Next, server $D^{i}$ determines that the tag group $G_{s}^{ox}$ is to be transferred to the multi-owner set $R^{j-oy}$. It first verifies that $R^{j-oy}$ is under the authority of $D^{i}$, obtains the secret value $K_{TTP}^{ox}$ that is used to generate the communication key of all individual tags $G_{sleaf}^{ox}$ in tag group $G_{s}^{ox}$, and uses this secret value to encrypt each tag ID $TID_{v}^{ox}$ to update the communication key of the new owner’s back-end server.

In Step 11, after server $D^{i}$ of the old owners receives messages $M_{8}$, it decrypts the message by using the communication key $SK_{D\_TTP}^{i}$ shared with the TTP and determines that $G_{s}^{ox}$ is part of the transfer message of the requesting owner $R_{1}^{i}$ in $R^{i-ox}$. The server then uses group communication key $GK_{s}^{ox}$ to encrypt the OT, $M_{5}$, $M_{7}$, and group ID $G_{s}^{ox}$ into message $M_{9}$. Subsequently, it uses the communication key $SK_{1}^{i}$ shared with owner $R_{1}^{i}$ to encrypt message $M_{9}$ into message $M_{10}$, which is then sent to owner $R_{1}^{i}.$

In Step 12, after the owner $R_{1}^{i}$ receives messages $M_{10}$, it decrypts the message by using the communication key $SK_{1}^{i}$ shared with server $D^{i}$ to extract message $M_{9}$, which is sent to tag group $G_{s}^{ox}$ as a broadcast message. When any tag receives message $M_{9}$, it confirms whether the group tag ID in the message matches the ID $G_{s}^{ox}$ of the tag group to which it belongs. After confirming that it belongs to the tag group $G_{s}^{ox}$, each tag uses the communication key $Kt_{v}^{ox}$ shared with server $D^{i}$ to decrypt and compare the OT. Tag $TID_{v}^{ox}$ uses message $M_{5}$ to calculate the secret value $K_{TTP}^{ox}$ that is used to generate the communication key of all individual tags in tag group $G_{s}^{ox}$. Next, the tag uses the calculated secret value $K_{TTP}^{ox}$ to decrypt message $M_{7}$, checks whether tag group $G_{s}^{ox}$ matches the claimed tag group ID, and then checks whether the random numbers $N_{r}$ are identical (if they are identical, then they represent the same communication). After authentication, the secret value $K_{TTP}^{ox}$ is used to encrypt tag ID $TID_{v}^{ox}$ to replace tag $TID_{v}^{ox}$ and the communication key of the back-end server. When all tags in tag group $G_{s}^{ox}$ are completely updated, the ownership of this group has been transferred from the multi-owner set $R^{i-ox}$ to the set $R^{j-oy}$.

### 3.4. Group Update and Balancing of the Key Tree

When implementation of the ownership transfer protocol is complete, the shared key on each transferred tag has been updated to the key shared with the back-end server of the new multi-owner set. Therefore, the server of the old owners can no longer be updated. However, a group communication key has yet to be established. When a member joins or leaves a group, the group communication key must be updated, and the balance state of the tree architecture must be checked. After the tag group architecture has been reconstructed in the server, existing methods for updating the group communication key, such as the approach proposed by Xu et al. [34], are used to update the communication key shared between groups.

## 4. Security Analysis

The method proposed in this study assumes that, after the mobile reader and back-end server authenticate each other by using an existing network security architecture, a shared communication key can be used in the subsequent protocol to identify the message deliverer and that, during communication, a shared communication key can be used for encryption to ensure secure communication. Hence, the following sections provide a security analysis of confidentiality between the tag and mobile reader, anti-replay attack, anti–man-in-the-middle attack, forward and backward secrecy, the windowing problem, location privacy protection, and an anti-DoS attack.

*A.*  *Confidentiality*

Communication between a mobile reader and a back-end server is encrypted using a shared communication key to ensure secure communication. Communication between a tag and mobile reader is encrypted using a communication key shared by the tag and the back-end server and a management key shared by the tag and the TTP. Attackers cannot decrypt the encrypted messages and, thus, cannot access the communicated information.

*B.*  *Anti-Replay Attack*

A random number and the communicated message are collectively encrypted so that, during the process of communication at all stages of the protocol, the sent message read by each tag changes, according to the random number. In step 1, if the attacker resends *M_1_*, the server will easily find it after decryption. This prevents attackers from completing authentication by replaying the previously acquired message.

*C.*  *Anti–Man-in-the-Middle Attack*

Communication among a mobile reader, tags, and the back-end server is encrypted, and a communication key shared among these three entities is used to confirm identity in order to proceed with ownership transfer. Additionally, because attackers do not have a shared key and cannot complete authentication through replay attacks, attackers cannot counterfeit the reader or tag to implement a man-in-the-middle attack.

*D.*  *Forward Secrecy*

In the protocol, the authentication and communication encryption key currently used by a tag are not given to the new owner. Instead, the new owner receives a new communication key, which is derived from a tag ID encrypted with a secret value that is randomly generated by the TTP. Therefore, the new owner cannot obtain the tag’s original communication key to decrypt any messages that were encrypted previously using the tag’s original key. Thus, forward secrecy is achieved.

*E.*  *Backward Secrecy*

After the new owner acquires the new communication key of a tag, the TTP updates the key, which is shared between the old owner and the tag, through the old owner. Because the old owner’s server and mobile reader have no access to the management key shared by the tags and the TTP, the old owner cannot access the secret value, which is randomly generated by the TTP and used to generate a new communication key. Therefore, the old owner cannot continue to track the tag’s subsequent information. Thus, backward secrecy is achieved.

*F.*  *Windowing Problem*

Because the old owner’s server and mobile reader have no access to the management key shared by the tags and the TTP, the old owner cannot access the secret value, which is randomly generated by the TTP and used to generate a new communication key. Moreover, neither the old owner nor attackers can successfully update the tag’s key by replaying the update-key message in the previous stage. This approach, thus, prevents the windowing problem in which both old and new owners hold tag ownership.

*G.*  *Location Privacy Protection*

In the protocol, the mobile reader is only responsible for sending out a message because a mobile reader in a mobile RFID environment may be a malicious attacker. Therefore, the message sent between a tag and the back-end server is encrypted along with a random number. Thus, the attacker cannot track the tag by analyzing the content of the message sent between the mobile reader and tag or by analyzing the messages sent at different stages between the mobile reader and tag. In other words, the method proposed in the present study can protect the tag’s location privacy.

*H.*  *Asynchronous Denial-of-Service Attack*

Asynchronous DoS attack on the back-end server and tags may occur when a message is lost or when attackers maliciously block a message. In the proposed protocol, the back-end server retains the tags’ keys before and after they are updated. Therefore, if the update-key message is lost or maliciously blocked, then the tag’s pre-updated key can be used to decrypt the message sent by a tag, which enables the owner to read the message. This is otherwise prevented when the server and tag become asynchronized.

Table 3 presents a comparison of the proposed protocol and other ownership transfer methods in terms of the following security concerns: forward secrecy (FS), backward secrecy (BS), replay attack (RA), DoS attack, the windowing problem (WP), and group ownership transfer (GO). In Table 3, “V” represents a secure protocol and “X” denotes an unsecure protocol.

## 5. Performance Analysis

This section details the analysis conducted in the present study on the calculation and information load of the proposed ownership transfer method. In this study, the proposed method is compared with other methods by analyzing and comparing the calculation load required to transfer the ownership of *n* tags belonging to old owners *os* to new owners *ns*. *T_E_* represents the amount of time required to calculate encryption and decryption once. *T_LE_* represents the amount of time required to calculate lightweight encryption and decryption once. *T_RNG_* represents the amount of time required to generate a random number, and *T_H_* represents the amount of time required to compute a hash function once.

The following aspects were compared between the method proposed in the present study and those proposed by Kapoor et al. [26] and Sundaresan et al [27,28]: the calculation load of tags, readers, servers, and the information load of the whole protocol. In the multi-owner single-tag ownership transfer method developed by Kapoor et al., encryption/decryption, hash functions, exclusive or (XOR), and random numbers are used. In their protocol, tags are transferred individually. To transfer a large number of tags, the protocol must be implemented multiple times, which results in decreased efficiency. Similarly, the multi-owner multi-tag ownership transfer method presented by Sundaresan et al. uses only pseudorandom number generator (PRNG), XOR, and random numbers. However, in their protocol, the group key shared among the server, the tag group, and the owners is used to individually generate transfer request messages for the transfer of each tag. Consequently, transfer efficiency is affected by the number of owners and tags. The method proposed in the present study uses *k*-ary to generate a tag group key tree architecture, which leads to different situations when transferring ownership.

**Figure 6:**
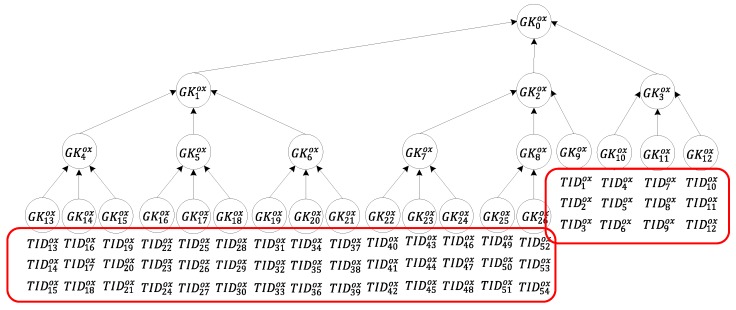
Tag transfer in Situation (1).

Situation (1): the whole group of tags is transferred, as illustrated in Figure 6. In this situation, the server only requires one set of group keys $GK_{0}^{ox}$ to notify all tags of the transfer request. Regarding the calculation, each tag only requires encryption/decryption to be performed six times. The reader, in order to send the message to all tags and read the message returned by each tag to the server, only needs to perform multi-owner confirmation once and message broadcasting once. The server is only required to perform the calculation once.

**Figure 7:**
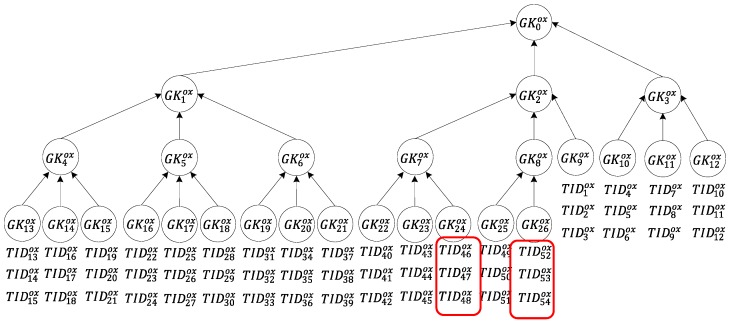
Tag transfer in Situation (2).

Situation (2): Only two tag groups are transferred, as shown in Figure 7. In this situation, the server requires two sets of group keys $GK_{24}^{ox}$ and $GK_{26}^{ox}$ to notify all tags of the transfer request. Regarding calculation, each tag is only required to conduct encryption/decryption computation six times. The reader, in order to send a message to all tags to be transferred and read the message that is returned by each of these tags to the server, is only required to perform multi-owner confirmation once and message broadcasting twice. The server is only required to perform calculation twice.

**Figure 8:**
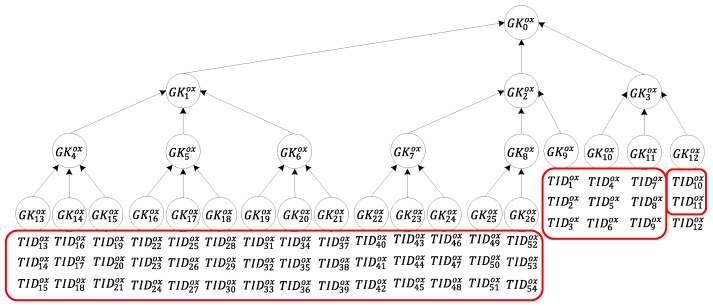
Tag transfer in Situation (3).

Situation (3): Only a single tag is not transferred, as depicted in Figure 8. In this situation, the server requires four sets of group keys and two sets of tag keys $GK_{1}^{ox}$, $GK_{2}^{ox}$, $GK_{10}^{ox}$, $GK_{11}^{ox}$, $Kt_{10}^{ox}$, and $Kt_{11}^{ox}$ to notify the tags that are to be transferred of the transfer request. Regarding calculation, each tag is only required to conduct encryption/decryption computation six times, and the reader, in order to send a message to all tags to be transferred and read the message that is returned by each of these tags to the server, is only required to perform multi-owner confirmation once and message broadcasting six times. The server only needs to perform the calculation six times.

The calculation load of the proposed ownership transfer method is indicated in Table 4, which provides an analysis of all components participating in the ownership transfer process. Compared with the encryption method, XOR operation and comparison operation exhibit a lighter calculation load and, thus, are negligible. In Table 4, message encryption and decryption are assumed to be performed through one calculation. The server calculates *T_RNG_* once for each key produced.

**Figure 9:**
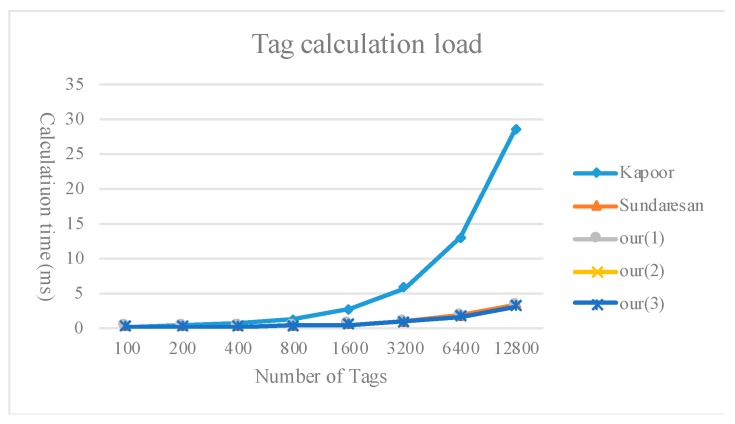
Comparison of the tag calculation load.

**Figure 10:**
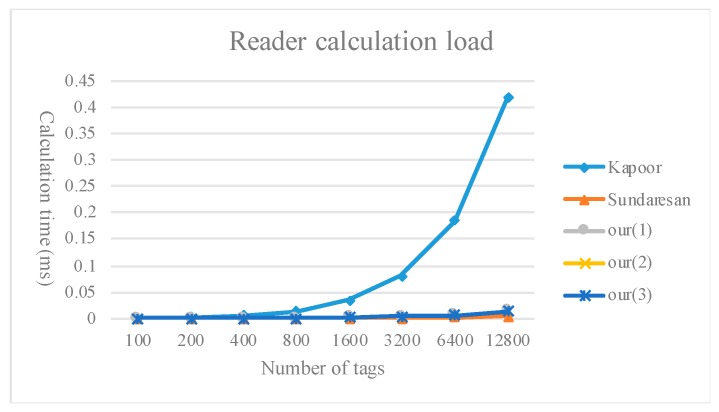
Comparison of the reader calculation load.

**Figure 11:**
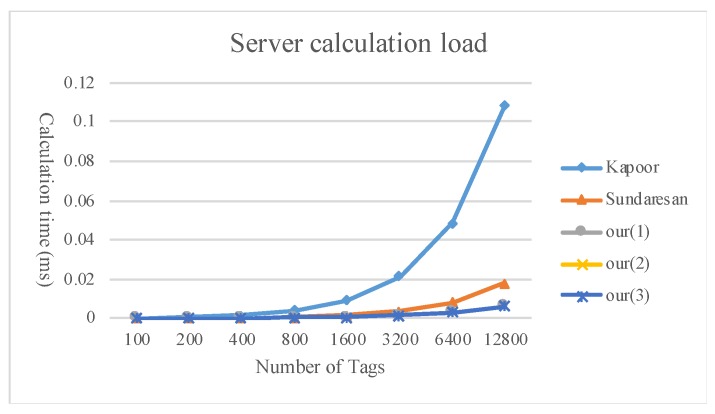
Comparison of the server calculation load.

Figure 9, Figure 10 and Figure 11 compare the calculation load of the tags, readers, and servers between the method proposed in the present study and existing multi-owner ownership transfer methods.

## 6. Conclusions

The present study proposes a secure, high-performance multi-owner partial tag ownership transfer protocol. A threshold scheme is adopted to ensure that the consent of a specific number of old owners is obtained before the ownership of a tag group can be partially transferred to new owners. This study introduces a method that can transfer the ownership of one, some, or all tags. The calculation load of the tags, readers, and corresponding servers was analyzed and compared with those of other multi-owner ownership transfer protocols. The results verified the high performance of the proposed protocol. Additionally, compared with other multi-owner ownership transfer methods, the proposed protocol is effectively more immune to RFID-defined attacks, such as eavesdropping, replay, man-in-the-middle, and DoS attacks. With the proposed protocol, the owners at the front end of a supply chain can transfer their ownership securely and efficiently.

## Figures and Tables

**Table 1:** Categories of RFID ownership transfer protocols.

Category	Protocols
Single owner/single tag	Osaka et al. [6], Fouladgar et al. [8], Taqieddin et al. [9], Kapoor et al. [10], Jäppinen et al. [11], Zuo [12], Yoon et al. [13], Chen et al. [14], Yang et al. [15], Dimitriou et al. [16], Kapoor et al. [18], and Yang [19].
Single owner/multiple tags	He et al. [20], Jannati et al. [21], Molnar et al. [22], and Tsai et al. [23]
Single owner/partial tags	Yang et al. [24,25].
Multiple owners/multiple tags	Kapoor et al. [26], Sundaresan et al. [27,28], and Munilla et al. [29].

**Table 2:** Notations.

Symbol	Description
$DID^{i}$	ID of owner server $\;D^{i}$.
$R_{}^{i-ox}$, $R_{}^{j-oy}$	Multi-owner set belong to server $D^{i}$ and $D^{j}$
$R_{1}^{i}$	One of the owners belonging to $\;R_{}^{i-ox}.$
$TID_{v}^{ox}$	*v*th tag ID owned by $\;R_{}^{i-ox}$.
$G_{s}^{ox}$	*s*th group ID owned by $\;R_{}^{i-ox}$.
$GK_{s}^{ox}$	Group key of group ID $\;G_{s}^{ox}\;$ and server $\;D^{i}$.
$Kt_{v}^{ox}$	Key shared between tag ID $TID_{v}^{ox}\;$ and server $\;D^{i}$.
$K_{v}^{ox}$	Management key shared between tag ID $TID_{v}^{ox}\;$ and the TTP.
$SK_{p}^{i}$	Key shared between the two entities *i* and *p.*
$K_{TTP}^{ox}$	Secret value shared between the tag group and the TTP.
$N_{r}$	Random number generated by the readers of multiple old owners.
OT	Ownership transfer request.
||	Signal connection notation.
E(key, msg)	Symmetric keyed encryption/decryption function, which uses a key to encrypt and decrypt a message.
LE(key, msg)	Lightweight symmetric keyed encryption/decryption function, which uses a key to encrypt and decrypt a message.

**Table 3:** Comparison of security.

	Kapoor et al. [26]	Sundaresan et al. [27,28]	Our Protocol
Forward secrecy (FS)	Ⅴ	Ⅹ	Ⅴ
Backward secrecy (BS)	Ⅴ	Ⅹ	Ⅴ
Replay attack (RA)	Ⅹ	Ⅹ	Ⅴ
Denial of service attack (DoS)	Ⅹ	Ⅹ	Ⅴ
Windowing problem (WP)	Ⅴ	Ⅴ	Ⅴ
Group ownership transfer (GO)	Ⅹ	Ⅴ	Ⅴ

**Table 4:** Performance comparison.

	Device	Calculation Load	Information Load
Kapoor et al. [26]	Tag	$\left(ns+1\right)T_{LE}+\left(ns+1\right)T_{H}+2T_{RNG}$	os+4ns+2
	Reader	$\left(os+ns\right)T_{E}+nsT_{LE}+2nsT_{H}+nsT_{RNG}$	
	Server	$\left(os+ns\right)T_{E}+T_{LE}+\left(ns+1\right)T_{H}+3T_{RNG}$	
Sundaresan et al. [27,28]	Tag	$\left(5n+3ns\right)T_{RNG}$	9ns+5n+(2n)ns
	Reader	$\left(6ns+2n\right)T_{RNG}$	
	Server	$\left(9ns+4n+\left(2n\right)ns+4\right)T_{RNG}$	
Our protocol (1)	Tag	$6nT_{LE}$	2os+2n+8
	Reader	$\left(2os+n+3\right)T_{E}+T_{RNG}$	
	Server	$\left(2os+n+9\right)T_{E}+\left(3n+3\right)T_{LE}+T_{RNG}$	
Our protocol (2)	Tag	$6nT_{LE}$	2os+2n+15
	Reader	$\left(2os+n+5\right)T_{E}+T_{RNG}$	
	Server	$\left(2os+n+17\right)T_{E}+\left(3n+6\right)T_{LE}+2T_{RNG}$	
Our protocol (3)	Tag	$6nT_{LE}$	2os+2n+43
	Reader	$\left(2os+n+13\right)T_{E}+T_{RNG}$	
	Server	$\left(2os+n+49\right)T_{E}+\left(3n+18\right)T_{LE}+6T_{RNG}$	

## References

[1] Juels A. (2006). RFID security and privacy: A research survey. IEEE J. Sel. Areas Commun..

[2] Seidler C. (2005). RFID Opportunities for Mobile Telecommunication Services, ITU-T Lighthouse Technical Paper. http://www.itu.int/ITU-T/techwatch/rfid.pdf.

[3] Lee H., Kim J. Privacy threats and issues in mobile RFID. Proceedings of the First International Conference on Availability, Reliability and Security (ARES’06).

[4] Engberg S.J., Harning M.B., Jensen C.D. Zero-knowledge Device Authentication: Privacy & Security Enhanced RFID preserving Business Value and Consumer Convenience. Proceedings of the Second Annual Conference on Privacy, Security and Trust.

[5] Van Deursen T., Mauw S., Radomirović S., Vullers P. Secure ownership and ownership transfer in RFID systems. Proceedings of the European Symposium on Research in Computer Security.

[6] Osaka K., Takagi T., Yamazaki K., Takahashi O. (2008). An efficient and secure RFID security method with ownership transfer. RFID Security.

[7] Juels A., Pappu R. (2005). RFID privacy: An overview of problems and proposed solutions. IEEE Secur. Priv..

[8] Fouladgar S., Afifi H. An efficient delegation and transfer of ownership protocol for RFID tags. Proceedings of the First International EURASIP Workshop on RFID Technology.

[9] Taqieddin E., Al-Dahoud H., Niu H., Sarangapani J. (2018). Tag Ownership Transfer in Radio Frequency Identification Systems: A Survey of Existing Protocols and Open Challenges. IEEE Access.

[10] Kapoor G., Piramuthu S. (2010). Vulnerabilities in Some Recently Proposed RFID Ownership Transfer Protocols. IEEE Commun. Lett..

[11] Jäppinen P., Hämäläinen H. Enhanced RFID security method with ownership transfer. Proceedings of the 2008 International Conference on Computational Intelligence and Security.

[12] Zuo Y.J. Changing Hands Together: A Secure Group Ownership Transfer Protocol for RFID Tags. Proceedings of the 2010 43rd Hawaii International Conference on System Sciences.

[13] Yoon E.J., Yoo K.Y. Two Security Problems of RFID Security Method with Ownership Transfer. Proceedings of the 2008 IFIP International Conference on Network and Parallel Computing.

[14] Chen H.B., Lee W.B., Zhao Y.H., Chen Y.L. Enhancement of the RFID Security Method with Ownership Transfer. Proceedings of the 3rd International Conference on Ubiquitous Information Management and Communication, SKKU.

[15] Yang X., Xu C., Li C. (2017). A privacy model for RFID tag ownership transfer. Secur. Commun. Netw..

[16] Dimitriou T. (2016). Key Evolving RFID Systems: Forward/Backward Privacy and Ownership Transfer of RFID Tags. Ad Hoc Netw..

[17] Lee C.C., Cheng C.L., Lai Y.M., Li C.T. Cryptanalysis of Dimitriou’s key evolving RFID systems. Proceedings of the Fifth International Conference on Network, Communication and Computing.

[18] Kapoor G., Piramuthu S. (2010). Single RFID tag ownership transfer protocols. IEEE Trans. Syst. Manand Cybern. Part C (Appl. Rev.).

[19] Yang M.H. (2011). Across-authority Lightweight Ownership Transfer Protocol. Electron. Commer. Res. Appl..

[20] He L.Y., Gan Y., Yin Y. (2014). Secure Group Ownership Transfer Protocol with Independence of Old Owner for RFID Tags. Comput. Model. New Technol..

[21] Jannati H., Falahati A. Cryptanalysis and enhancement of a secure group ownership transfer protocol for RFID tags. Proceedings of the Global Security, Safety and Sustainability & e-Democracy.

[22] Molnar D., Soppera A., Wagner D. A scalable, delegatable pseudonym protocol enabling ownership transfer of RFID tags. Proceedings of the Selected Areas in Cryptography.

[23] Tsai K.Y., Luo J.N., Yang M.H., Liew W.T. (2019). Novel designated ownership transfer with grouping proof. Appl. Sci..

[24] Yang M.H., Xie K.P. (2013). TTP-Based Group Ownership Transfer in A Mobile RFID Environment. Int. J. Digit. Content Technol. Its Appl..

[25] Yang M.H. (2012). Secure Multiple Group Ownership Transfer Protocol for Mobile RFID. Electron. Commer. Res. Appl..

[26] Kapoor G., Zhou W., Piramuthu S. (2011). Multi-tag and Multi-owner RFID Ownership Transfer in Supply Chains. Decis. Support Syst..

[27] Sundaresan S., Doss R., Zhou W. Secure ownership transfer in multi-tag/multi-owner passive RFID systems. Proceedings of the 2013 IEEE Global Communications Conference (GLOBECOM).

[28] Sundaresan S., Doss R., Zhou W., Piramuthu S. (2015). Secure Ownership Transfer for Multi-tag Multi-owner Passive RFID Environment with Individual-owner-privacy. Comput. Commun..

[29] Munilla J., Burmester M., Peinado A. (2016). Attacks on ownership transfer scheme for multi-tag multi-owner passive RFID environments. Comput. Commun..

[30] Poschmann A., Leander G., Schramm K., Paar C. New light-weight crypto algorithms for RFID. Proceedings of the 2007 IEEE International Symposium on Circuits and Systems.

[31] Ågren M., Hell M., Johansson T., Meier W. (2011). Grain-128 a: A new version of Grain-128 with optional authentication. Int. J. Wirel. Mob. Comput..

[32] Harn L. (1994). Group-oriented (t, n) threshold digital signature scheme and digital multisignature. IEEE Proc. Comput. Digit. Tech..

[33] Zhou J., Ou Y.H. Key tree and Chinese remainder theorem based group key distribution scheme. Proceedings of the International Conference on Algorithms and Architectures for Parallel Processing.

[34] Xu L., Huang C. (2008). Computation-efficient multicast key distribution. IEEE Trans. Parallel Distrib. Syst..

